# Digital Inequality During a Pandemic: Quantitative Study of Differences in COVID-19–Related Internet Uses and Outcomes Among the General Population

**DOI:** 10.2196/20073

**Published:** 2020-08-20

**Authors:** Alexander JAM van Deursen

**Affiliations:** 1 University of Twente Enschede Netherlands

**Keywords:** COVID-19, digital inequality, internet use, survey, personality, literacy, internet skills, information, communication

## Abstract

**Background:**

The World Health Organization considers coronavirus disease (COVID-19) to be a public emergency threatening global health. During the crisis, the public’s need for web-based information and communication is a subject of focus. Digital inequality research has shown that internet access is not evenly distributed among the general population.

**Objective:**

The aim of this study was to provide a timely understanding of how different people use the internet to meet their information and communication needs and the outcomes they gain from their internet use in relation to the COVID-19 pandemic. We also sought to reveal the extent to which gender, age, personality, health, literacy, education, economic and social resources, internet attitude, material access, internet access, and internet skills remain important factors in obtaining internet outcomes after people engage in the corresponding uses.

**Methods:**

We used a web-based survey to draw upon a sample collected in the Netherlands. We obtained a dataset with 1733 respondents older than 18 years.

**Results:**

Men are more likely to engage in COVID-19–related communication uses. Age is positively related to COVID-19–related information uses and negatively related to information and communication outcomes. Agreeableness is negatively related to both outcomes and to information uses. Neuroticism is positively related to both uses and to communication outcomes. Conscientiousness is not related to any of the uses or outcomes. Introversion is negatively related to communication outcomes. Finally, openness relates positively to all information uses and to both outcomes. Physical health has negative relationships with both outcomes. Health perception contributes positively to information uses and both outcomes. Traditional literacy has a positive relationship with information uses and both outcomes. Education has a positive relationship with information and communication uses. Economic and social resources played no roles. Internet attitude is positively related to information uses and outcomes but negatively related to communication uses and outcomes. Material access and internet access contributed to all uses and outcomes. Finally, several of the indicators and outcomes became insignificant after accounting for engagement in internet uses.

**Conclusions:**

Digital inequality is a major concern among national and international scholars and policy makers. This contribution aimed to provide a broader understanding in the case of a major health pandemic by using the ongoing COVID-19 crisis as a context for empirical work. Several groups of people were identified as vulnerable, such as older people, less educated people, and people with physical health problems, low literacy levels, or low levels of internet skills. Generally, people who are already relatively advantaged are more likely to use the information and communication opportunities provided by the internet to their benefit in a health pandemic, while less advantaged individuals are less likely to benefit. Therefore, the COVID-19 crisis is also enforcing existing inequalities.

## Introduction

### Background

The World Health Organization considers coronavirus disease (COVID-19) to be a public emergency threatening global health [1]. Governments worldwide have taken stringent action, including requiring social distancing, closing public services, schools and universities, and canceling cultural events [2,3]. People are being advised or ordered to stay at home and socially isolate themselves to avoid being infected [4]. The ongoing pandemic represents an outbreak of an unparalleled scale, and it has induced widespread fear and uncertainty.

In this paper, we focus on the role of the internet during the crisis. The internet has become a crucial source for the general public, as it provides access to general information, the latest national and international developments, and guidelines on behavioral norms during the crisis. In this respect, the internet plays an important role in the great challenges facing governments regarding the transfer of knowledge and guidelines to the population at large. When individuals understand the need and rationale behind government-enforced measures, they are more motivated to comply and even adopt measures voluntarily [5,6]. In addition to informational purposes, the internet enables individuals to share news and experiences with people they cannot meet face-to-face, remain in contact with friends and family, seek support, and ask questions of official agencies, including health agencies. Further, the internet enables people to take initiatives such as raising money or preparing packaged meals for people in need, such as health workers or people who have lost their jobs. In sum, the internet plays a vital role for people of all social strata and backgrounds during a time of worldwide crisis. All people should thus be able to use the internet as a source of information and communication.

**Figure 1:**
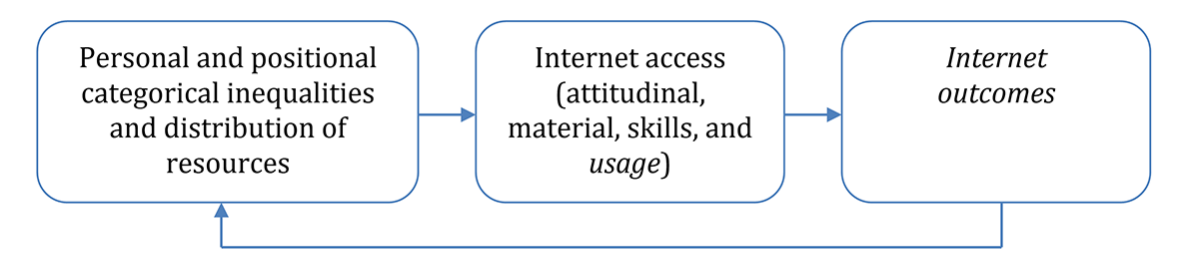
Simplified model of the resources and appropriation theory [8].

However, digital inequality research has shown that internet access is not evenly distributed among the general population [7,8]. The basic idea of digital inequality stems from a comparative perspective of social and information inequality, as there are benefits associated with internet access and negative consequences of lack of access [9]. Calamities are often a story of inequality [10]; therefore, in this paper, we aimed to gain a deeper and broader understanding of the differences in how people use the internet to cope during the COVID-19 crisis. Van Dijk’s resources and appropriation theory [8] explains differences or inequalities of internet access by considering personal and positional categories of individuals and the individuals’ resources. Internet access itself is considered to be a process of appropriation involving attitudinal access, material access, skills access, and in the final stage, usage access. The latter entails differences in the type of activities that people perform on the internet. The consequences of the process are the outcomes of internet use. These outcomes in turn reinforce personal and positional inequalities and an unequal distribution of resources [8] (Figure 1). The first goal of this paper is to provide a timely understanding of how different people use the internet and the outcomes they gain from it in relation to the COVID-19 pandemic.

Internet use and outcome differences between groups of people are likely to have profound consequences on how people manage a crisis. For example, older people are most in danger of being infected with the virus and most likely to die from the infection [11], and they also use the internet less and have the fewest internet outcomes [12]. The latter may further endanger their peculiar situation, as limited internet use and outcomes may result in a lack of critical information or necessary support.

### COVID-19–Related Internet Uses and Outcomes

To study differences in internet uses and outcomes during the COVID-19 pandemic, it is necessary to understand the types of uses and outcomes that are at play. Typically, uses and outcomes are studied by following conceptual classifications that distinguish different domains, such as economic, social, cultural, or personal domains [13]. Here, we take the COVID-19 pandemic as the domain of interest. Within this domain, we consider two main and conceptually different types of uses and outcomes: information and communication [14,15]. Information internet uses involve searching for information on all aspects of COVID-19. Potential information outcomes include becoming better informed about the disease, understanding why certain measures are necessary, and limiting the risk of becoming infected by developing greater awareness of one’s own behavior. Communication internet uses include talking to friends about the crisis, asking questions on social media or online fora, giving advice, or offering support to others. Communication outcomes include finding people on the internet who can offer support or share concern, being less lonely, and protecting others from potential COVID-19 risks. Studying both types of uses and outcomes is important, as prior research has shown that communication uses can compensate for information uses to attain beneficial internet outcomes [16].

### Determinants of COVID-19–Related Internet Uses and Outcomes

Digital inequality research suggests that the vast amount of web-based information and communication possibilities around the COVID-19 pandemic are likely to be difficult to grasp and conceptualize for sections of the general population [7]. Some frequently observed personal categorical inequalities are gender, age, personality, and health [7]. Earlier research revealed that men and women differ in their internet activities; women are more likely to use email and social media, whereas men are more likely to use the internet to obtain information [17,18]. Age in general has a negative impact on all types of internet uses and outcomes [7]. In the COVID-19 crisis, older people are especially vulnerable; therefore, it is very important for them to know how to behave and be safe. We hypothesize that (H1) men are more likely to be involved in information-related uses and outcomes while women are more likely to be involved in communication-related internet uses and outcomes regarding COVID-19-related internet uses and outcomes. We also hypothesize that (H2) age contributes negatively to COVID-19–related internet uses and outcomes.

An individual’s personality may hinder or stimulate their engagement in certain COVID-19–related activities. Cognitive appraisal theory suggests that individuals complete two types of cognitive appraisal processes in a crisis [19]. The process starts with an evaluation of the crisis as a potential source of danger or life disruption. If the crisis is not determined to be dangerous, it is not considered a stressor and does not require intervention. If the crisis is determined to be relevant, it is considered a stressor and must be evaluated further by balancing the demands of the crisis and the person’s resources [20]. At this point, personality enters the equation [20]. There is a general consensus regarding the Big Five model when personality traits are studied. This model proposes five personality traits of agreeableness, neuroticism, conscientiousness, introversion, and openness [21]. However, there is no agreement as to whether these traits contribute to or detract from resisting disturbance [20]. There is also no consensus on how the Big Five personality traits relate to internet use [7,22]. For example, conscientiousness relates to people who abide by rules. On one hand, one might argue that this would result in a greater need for information on how to behave. On the other hand, the internet is unstructured, and rules and policies are absent to a large extent. When linking personality traits to internet use for psychological adjustments to the COVID-19 crisis, it is not evident whether these traits will support or hinder COVID-19–related internet uses and outcomes. We hypothesize that (H3a) agreeableness, (H3b) neuroticism, (H3c) contentiousness, (H3d) introversion, and (H3e) openness are related to COVID-19–related internet uses and outcomes.

An individual’s health may play an important role in how they approach COVID-19. To gain an elaborate understanding of how health relates to COVID-19–related internet uses and outcomes, we followed earlier research that distinguishes between different health aspects [23]: A person’s physical functioning or the degree to which their health currently interferes with activities such as sports, carrying groceries, climbing stairs, and walking, their mental health or psychological distress and well-being, and their health perception concerning their own health rating in general. During a crisis, we expect that people with health issues are more likely to turn to the internet for comfort and reassurance. We hypothesize that (H4a) physical functioning, (H4b) mental health, and (H4c) health perception contribute negatively to COVID-19–related internet uses and outcomes.

The final type of personal inequality considered in this study is traditional literacy, which is known to have a substantial impact on how the internet is used [24,25]. We consider literacy to be the ability to read, write, and understand text, which is also framed under the umbrella terms functional literacy or fundamental literacy [24]. Functional or traditional literacy can be considered as the basic dimension of all literacy concepts [26]. Considering the crucial role the internet is playing in the COVID-19 crisis, a low level of literacy is a potentially large inhibitor of understanding information and being involved in web-based communication. We hypothesize that (H5) traditional literacy contributes positively to COVID-19–related internet uses and outcomes.

Education is the most observed positional categorial inequality in digital divide research, and it is likely to play a role in the current context. People with higher levels of education are better equipped to comprehend web-based information and benefit from internet use [7]. We hypothesize that (H6) education contributes positively to COVID-19–related internet uses and outcomes.

When studying differences in internet uses and outcomes, the resources people can access are often derived from Pierre Bourdieu’s capital theory [27], which stresses the importance of including not only economic but also social and cultural resources to determine one’s status and position in society. In the COVID-19 pandemic, economic and social resources are likely to be important, as earlier research has shown that people with greater economic resources—mostly operationalized as income in digital inequality research—are known to use the internet more efficaciously and productively [7,28]. People with more social resources are more likely to have access to family, friends, or other contacts on the internet [29]. We hypothesize that (H7a) economic and (H7b) social resources contribute positively to COVID-19–related internet uses and outcomes.

### The Internet Appropriation Process

The core of the resources and appropriation theory is access to technology, which is considered as a process of appropriation involving attitudinal, material, skills, and usage access. Attitudinal access concerns a person’s attitude towards the internet; according to theories of technology adoption, this type of access is crucial for using the internet [30]. Material access can be defined in terms of the different devices that people use to access the internet and all other web-based resources, including desktop computers, laptop computers, tablets, smartphones, game consoles, and interactive televisions [31]. Skills access concerns the skills necessary to use the internet, ranging from operational and information skills to social and content creation skills [32]. Prior research has revealed that all three types of internet access directly affect internet uses and outcomes [16]. We hypothesize that (H8a) attitudinal internet access, (H8b) material internet access, and (H8c) skills internet access contribute positively to COVID-19–related internet uses and outcomes.

### The Effects of COVID-19–Related Internet Uses on Their Corresponding Outcomes

A recent multifaceted consideration of digital inequality revealed a strong effect of internet uses on outcomes [12]. Further, people’s internet activities appeared to be more important than their personal characteristics with regard to inequalities in outcomes of internet use. This suggests that the variables discussed in the prior sections will become less important for obtaining information outcomes when people are involved in COVID-19–related internet information uses. This is also true for COVID-19–related communication uses and outcomes. The second goal of this paper is to reveal the extent to which the indicators discussed remain important for obtaining internet outcomes after people are involved in the corresponding uses.

## Methods

### Recruitment

This study used a web-based survey and drew upon a sample collected in the Netherlands. To obtain a representative sample of the population, we used PanelClix, a professional organization for market research, to provide a panel of approximately 110,000 people. Members of the panel received a small incentive for every survey they completed. In the Netherlands, 98% of the population uses the internet; therefore, the internet user population is very closely representative of the general population in terms of its sociodemographic makeup. The panel included novice and advanced internet users. In total, we aimed to obtain a dataset with approximately 1700 respondents over the age of 18. Eventually, this resulted in the collection of 1733 responses over a 1-week period in April 2020. During the data collection period, three amendments to the sampling frame were made to ensure the representativity of the Dutch population. Accordingly, the analyses revealed that the gender, age, and formal education of our respondents largely matched official census data. As a result, only very small post hoc corrections were needed.

The web-based survey used software that checked for missing responses and prompted users to respond. The survey was pilot-tested with 10 internet users over two rounds. Amendments were made based on the feedback provided. No major comments were provided in the second round. The average time required to complete the survey was 20 minutes.

### Measures

We initially developed 11 survey items pertaining to COVID-19–related internet use. Respondents were asked to indicate the extent to which they used the internet for various activities in the past month using a 5-point scale (“not” to “multiple times a day”) as an ordinal-level measure. Principal component analysis with varimax rotation was used to determine two underlying usage clusters, one related to information and one to communication. Factor loadings were employed at 0.4 and above for each item [33]. In total, 8 items (3 for information and 5 for communication) were retained in a two-factor structure with eigenvalues over 1.0, together accounting for 76% of the total variance.

For COVID-19-related information and communication internet outcomes, we developed 14 items mapped onto the use items. A 5-point agreement scale as an ordinal level measure was used. Principal component analysis with varimax rotation resulted in a structure that matched the conceptual definition of information outcomes (4 items) and communication outcomes (4 items). The two factors showed eigenvalues over 1.0 and explained 65% of the variance.

Gender was included as a dichotomous variable, and age was directly asked (mean 50.2, SD 17.0).

Personality was measured with the Quick Big Five personality questionnaire [34], which consists of 30 adjectives reflecting a valid and reliable measure of the Big Five traits. Participants were asked to rate the extent to which a particular adjective applied to them on a 7-point scale, ranging from completely untrue to completely true. The Cronbach α values for the five traits were .89 for agreeableness, .88 for neuroticism, .88 for conscientiousness, .87 for introversion, and .81 for openness.

Physical health, mental health, and health perception were measured with the Dutch version of the Medical Outcomes Study (MOS) Short-Form General Health Survey (SF-20) [35]. This instrument enables respondents to assess their general health and generates composite summary scores representing different types of health. We normalized the scales, with higher scores representing better functioning. Physical health was measured with 5 items (2-point scale; α=.89; mean 1.75, SD 0.34), mental health with 5 items (5-point scale; α=.85; mean 3.65, SD 0.77), and health perception with 5 items (5-point scale; α=.86; mean 3.39, SD 0.85).

To measure traditional literacy, we used the validated 11-item Diagnostic Illiteracy Scale [36]. Sample items included “I have difficulties with reading and understanding information from my municipality” and “I find it difficult to read and understand my telephone bill.” A 5-point agreement scale was used. Scores on the scale exhibited high internal consistency. Items were recoded so that higher scores corresponded with higher levels of literacy (α=.94; mean 4.33, SD 0.71).

To assess education, data regarding degrees earned were collected and used to create three groups: low (primary), middle (secondary), and high (tertiary) educational achievement.

Economic resources were objectively measured by seeking the annual family income in the last 12 months. Twelve categories were recoded into three categories of low for <€30,000 (US $35,503.50), middle for €30,000 to €70,000 (US $35,503.50 to $82841.50), and high for >€70,000 (>US $82841.50). For social resources, we used the MOS Social Support Survey [37]. Respondents completed 18 items covering emotional support (eg, “Someone you can count on to listen when you need to talk”), informational support (eg, “Someone to give you good advice about a crisis”), and tangible support (eg, “Someone to help you if you were confined to bed”). All items were rated on a 5-point Likert scale with anchors of none of the time (1) and most of the time (5). We computed an aggregate measure of support availability (α=.96; mean 3.83, SD 0.85).

Attitudinal internet access was measured by three items adapted from the Digital Motivation Scale [38]. A 5-point agreement scale was used, and all items were balanced for the direction of response (α=.74; mean 4.10, SD 0.70). An example statement is “Technologies such as the internet and mobile phones make life easier.” To measure material internet access, we considered 7 devices used to connect to the internet (mean 3.43, SD 1.53). Included were desktop computer, laptop computer, tablet, smartphone, smart TV, game console, and smart device (eg, activity tracker). Finally, skills internet access was adapted from the conceptual idea behind the Internet Skills Scale [32]. We proposed 30 items reflecting operational, information navigation, social, and creative internet skills. A 20-item single skills construct resulted from the principal component analysis. All items were scored on a 5-point scale that ranged from “not at all true of me” to “very true of me” and exhibited high internal consistency (α=.96; mean 3.67, SD 0.97). Example items are “I know how to open downloaded files,” “I find it hard to decide what the best keywords are to use for online searches,” and “I know which information I should and shouldn’t share online.”

### Statistical Analysis

To test the hypotheses and account for the sequentiality between COVID-19–related internet uses and outcomes, hierarchical regression analyses were used. In the first model, we tested our hypotheses by analyzing the significant determinants for the two types of COVID-19–related internet uses and the two corresponding outcomes. In the second model, we sought to determine the changes in the significance of the determinants after the internet uses were added to the models.

## Results

Table 1 provides an overview of the sample of people surveyed in the study.

Table 2 shows the mean scores of the survey questions related to internet uses and internet outcomes.

The first goal of this paper was addressed in the first model, as presented in Table 3, where several significant determinants for COVID-19 uses and outcomes are revealed.

**Table 1:** Demographic profile of the Dutch internet user sample (N=1733), n (%).

Characteristic		Value
**Gender**		
	Male	874 (50.4)
	Female	859 (49.6)
**Age (years)**		
	18-30	280 (16.2)
	31-40	271 (15.6)
	41-50	293 (16.9)
	51-60	338 (19.5)
	61-70	324 (18.7)
	>70	227 (13.1)
**Education level^a^**		
	Low	519 (29.9)
	Middle	602 (34.7)
	High	612 (35.3)

^a^Low: primary; middle: secondary; high: tertiary.

**Table 2:** Survey questions and responses on the 5-point Likert scale.

Category and questions		α	Mean (SD)
**COVID-19^a^–related informational internet uses**		.80	3.13 1.53
	Search the internet for information about COVID-19		3.76 1.91
	Consult websites of public agencies (eg, RIVM^b^, municipality, hospital, or government)		3.21 1.83
	Search the internet for measures to prevent the further spread of COVID-19		2.44 1.71
**COVID-19–related communication internet uses**		.92	1.56 1.13
	Provide advice on COVID-19 to others via social media		1.56 1.31
	Starting an action against COVID-19 via the internet (eg collecting money, offering help)		1.41 1.17
	Ask questions about COVID-19 on forums or social media		1.54 1.30
	Comment on the internet on COVID-19 discussions (eg, on social media)		1.58 1.34
	Offering help online to people who need it now		1.70 1.41
**COVID-19–related information internet outcomes**		.80	3.17 0.95
	The internet makes me better informed about COVID-19		3.58 1.13
	The internet makes me understand the measures against COVID-19 better		3.25 1.15
	The internet helps me to reduce the risk of getting COVID-19		3.15 1.16
	Information about COVID-19 on the internet has made me more aware of my own behavior		2.70 1.26
**COVID-19–related communication internet outcomes**		.80	1.91 0.89
	Through the internet I found someone who can help me in this time of COVID-19		1.67 1.04
	Through the internet I have found people with whom I can share my concerns about COVID-19		1.83 1.10
	Via the internet I contributed to the COVID-19 crisis (eg, collecting money, helping people)		1.83 1.13
	The internet makes me less lonely now		2.29 1.25

^a^COVID-19: coronavirus disease.

^b^RIVM: Rijksinstituut voor Volksgezondheid en Milieu.

**Table 3:** Hierarchical regression analysis summary for coronavirus disease–related internet uses and outcomes (Model 1).

Characteristic		Information				Communication			
		Use		Outcome		Use		Outcome	
		β	*P* value	β	*P* value	β	*P* value	β	*P* value
**Gender and age**									
	Gender (male or female)	.01	.61	.00	.98	–.08	<.001	.01	.83
	Age	.08	.01	–.03	.35	–.08	.003	–.11	<.001
**Big Five personality traits**									
	Agreeableness	-.07	.03	–.01	.75	–.13	<.001	-.08	.003
	Neuroticism	.15	<.001	.15	<.001	.05	.20	.08	.02
	Conscientiousness	.01	.60	–.02	.52	.01	.54	–.04	.14
	Introversion	–.04	.11	.02	.56	–.09	<.001	-.06	.02
	Openness	.08	.004	.03	.30	.14	<.001	.15	<.001
**Health status**									
	Physical health	–.04	.15	–.03	.31	–.15	<.001	–.10	<.001
	Mental health	–.06	.15	.03	.41	–.06	.11	–.01	.82
	Health perception	.07	.05	.04	.31	.16	<.001	.10	<.001
**Literacy and education**									
	Traditional literacy	.09	<.001	.10	.18	.31	<.001	.33	<.001
	Education	.08	.002	.02	.36	.07	.003	.02	.34
**Resources**									
	Economic resources	.03	.23	.04	.13	–.01	.57	–.03	.23
	Social resources	.02	.40	.00	.91	–.02	.36	–.01	.69
**Access**									
	Attitudinal access	.14	<.001	.29	<.001	–.06	.01	–.04	.08
	Material access	.10	<.001	.06	.02	.08	<.001	.07	.008
	Skills access	.08	.006	.08	.008	.09	<.001	.09	<.001

Table 3 shows that men are more likely to be involved in COVID-19–related communication uses. Age is positively related to COVID-19–related information uses and negatively related to COVID-19 communication uses and outcomes. Concerning personality traits, agreeableness is negatively related to COVID-19–related information and communication uses and to communication outcomes. Neuroticism is positively related to both uses and to communication outcomes.

Conscientiousness is not related to any of the uses or outcomes. Introversion is negatively related to COVID-19–related communication uses and outcomes, suggesting that this is performed more by extraverted people. Finally, openness relates positively to information uses and to both outcomes.

The results further show that concerning the three health indicators, physical health is negatively related to communication uses and outcomes. Mental health did not contribute to any uses or outcomes. Health perception contributes positively to information uses and to both outcomes.

Traditional literacy has a positive relationship with information-type uses and with both outcomes, and education has a positive relationship with COVID-19–related information and communication uses. Economic and social resources were not related to any COVID-19 uses or outcomes.

Attitudinal internet access is positively related to information uses and outcomes but is negatively related to communication uses and outcomes. Material internet access contributes positively to all uses and outcomes, and skills access has a positive relationship with all uses and outcomes. Table 4 provides an overview of the hypotheses.

**Table 4:** Overview of the hypotheses.

Number	Hypothesis	Information uses	Information outcomes	Communication uses	Communication outcomes	Validation
H1	Gender (male or female)	ns^a^	ns	–^b^	ns	R^c^
H2	Age	+^d^	ns	–	–	PS^e^
H3a	Agreeableness	–	ns	–	–	PS
H3b	Neuroticism	+	+	ns	+	PS
H3c	Conscientiousness	ns	ns	ns	ns	R
H3d	Introversion	ns	ns	–	–	PS
H3e	Openness	+	ns	+	+	PS
H4a	Physical health	ns	ns	–	–	PS
H4b	Mental health	ns	ns	ns	ns	R
H4c	Health perception	+	ns	+	+	R
H5	Traditional literacy	+	ns	+	+	PS
H6	Education	+	ns	+	ns	PS
H7a	Economic resources	ns	ns	ns	ns	R
H7b	Social resources	ns	ns	ns	ns	R
H8a	Attitudinal access	+	+	–	–	PS
H8b	Material access	+	+	+	+	S^f^
H8c	Skills access	+	+	+	+	S

^a^ns: no significant contribution.

^b^–: significant negative contribution.

^c^R: reject.

^d^+: significant positive contribution.

^e^PS: partial support.

^f^S: support.

Finally, to address the second goal of the study, we tested what would happen to the contribution of the outcome determinants when the corresponding uses were added to the analyses (Model 2: see Tables 5 and 6). Adding the uses significantly increased the explained variance; also, several of the relationships between personal and positional categories and between resources and outcomes became insignificant. The relationships that remained significant for information outcomes were age, health perception, and traditional literacy. Furthermore, attitudinal internet access remained significant. For communication outcomes, the relationships that remained significant were age, openness, and traditional literacy.

**Table 5:** Hierarchical regression analysis summary for coronavirus disease–related internet outcomes (Model 2).

Characteristic		Information outcomes		Communication outcomes	
		β	*P* value	β	*P* value
**Gender and age**					
	Gender (male or female)	–.01	.71	.04	.05
	Age	–.07	.003	–.08	<.001
**Big Five personality traits**					
	Agreeableness	.03	.25	–.02	.38
	Neuroticism	.06	.04	.06	.06
	Conscientiousness	–.02	.26	–.04	.05
	Introversion	.04	.08	–.02	.40
	Openness	-.02	.49	.08	<.001
**Health status**					
	Physical health	.07	.05	–.03	.26
	Mental health	.01	.79	.02	.59
	Health perception	–.05	.02	.03	.34
**Literacy and education**					
	Traditional literacy	.05	.02	.19	<.001
	Education	–.02	.33	–.01	.67
**Resources**					
	Economic resources	.02	.30	–.02	.28
	Social resources	–.01	.67	.00	.99
**Access**					
	Attitudinal access	.21	<.001	–.02	.49
	Material access	.01	.74	.03	.21
	Skills access	.03	.17	.05	.06
Information uses		.55	<.001	N/A^a^	N/A
Communication uses		N/A	N/A	.45	<.001

^a^N/A: not applicable.

**Table 6:** Changes in the significance of the determinants after internet uses were added to the models (*P*<.001).

Model and measures		Information outcomes		Communication outcomes	
		Use	Outcome	Use	Outcome
**Model 1**					
	r^2^	.09	.13	.23	.21
	F	22.15	15.05	30.13	26.54
**Model 2**					
	r^2^	N/A^a^	.41	N/A	.37
	r^2^ change	N/A	.28	N/A	.16
	F	N/A	63.71	N/A	54.72

^a^N/A: not applicable.

## Discussion

### Principal Results

This paper aims to provide a comprehensive examination of digital inequality in the case of an unprecedented health pandemic. The first goal of the study was to reveal how inequality manifests itself in COVID-19–related internet information and communication uses and outcomes. The findings revealed several relationships between the background variables and the two types of internet uses and outcomes.

Older people were found to be less equipped to use the internet for information and communication during a time of crisis. However, they were more likely to engage in information-type COVID-19–related internet uses, possibly because they are at greatest risk from the disease [11]. This did not result in more beneficial information outcomes. Internet skills play an important role in translating internet uses into beneficial internet outcomes [39], and prior research has shown that older people have lower internet skill levels in general [32]. The finding that older people are less likely to perform communication activities or obtain communication-related outcomes is in line with prior studies [15]; however, these outcomes are important, as older people are more at risk of having severe complications when diagnosed with COVID-19. Regarding gender, contrary to general internet use, men were found to be more likely to engage in communication-type COVID-19–related internet uses during the crisis than women. A possible explanation is that men and women may respond to crisis news in different ways [40].

The positive effect of neuroticism suggests that a tendency to experience negative emotions such as anger, anxiety, or depression fuels the need to turn to the internet for COVID-19–related information and communication. People who score higher on the neuroticism scale may be more in need of guidelines on how to mitigate risks or may need more support from others to be comforted. Also, the openness trait supports both information and communication internet use and outcomes. A possible explanation is that a major crisis triggers adventure, unconventional ideas, imagination, awareness of feelings, curiosity, or a variety of experiences, all of which are aspects linked to high openness [21]. The negative contribution of agreeableness raises questions. A possible explanation is that agreeable people are less frequently sought out for communication activities. However, the internet may also be a very inviting environment for less agreeable people. Conscientiousness did not appear to be a significant determinant. People who are more stubborn and focused or more flexible and spontaneous both appear to be involved in information- and communication-type COVID-19–related internet uses and outcomes. Extroversion emerged as a trait that supports using the internet for communication uses and outcomes; this can be expected, as extroversion is marked by pronounced engagement with the external world [21].

Although we expected that psychological distress would play a role in the current context, as there would be a relatively high need for information and support from others, mental health did not surface as a significant contributor. Furthermore, we did find that physical health problems appear to encourage web-based COVID-19–related communication uses and outcomes. The most likely explanation is that people with underlying health problems are more at risk (and thus more bound to their homes) and thus have higher needs for communication with friends and family. A possible reason for the positive effect of health perception is that people who believe their personal health to be good may feel better equipped to support others during the COVID-19 pandemic.

As expected, traditional literacy played an important role. A lack of general ability to read, write, and understand text further disadvantages individuals in the case of the COVID-19 pandemic, as they have less access to information and communication sources. COVID-19 is a new, unknown, and complicated disease with characteristics that are often described in difficult medical language that is not easy to read. Similar findings were found for educational attainment. Research has long shown that education is one of the most prominent positional variables in digital divide research [7]. However, our results suggest that when less educated individuals are involved in information and communication internet uses, they are as likely to achieve the corresponding outcomes as people with higher levels of education. This is an important finding for designing interventions for those of lower levels of education.

An effect of economic resources did not emerge in relation to COVID-19–related internet uses and outcomes. The participants’ income did not make a difference in obtaining information and communication COVID-19–related internet outcomes. Earlier research often showed that income is especially important to consumptive and work-related internet uses [17], topics that are not considered here. Unexpectedly, social resources were not found to be influential. Apparently, a person who has an offline support network will not necessarily turn more to web-based information and communication support during a crisis.

Concerning internet access, we can first conclude that a person’s internet attitude is important for engaging in information uses and gaining information outcomes. Unexpectedly, there was a negative contribution of internet attitude to communication uses and outcomes, suggesting that individuals who have a negative evaluation of the internet in general are more likely to engage in communication uses in the event of a major crisis. Both material and skills internet access played important roles in achieving all uses and outcomes. Using a higher diversity of devices was related to higher COVID-19–related internet use and to more outcomes. The opportunities devices offer are known to be related to inequalities in internet uses and outcomes. As each device offers its own specific characteristics and advantages, a higher diversity of devices supports a larger range of use activities and outcomes [31]. Furthermore, internet skills play a fundamental role in COVID-19–related uses and in obtaining beneficial outcomes [12].

In this paper, several indicators surfaced for people’s web-based COVID-19–related uses and outcomes. The variety of important indicators raises the question of whether general policies to address digital inequalities in a time of crisis will be effective. The complex relationships between the different indicators on one hand and internet uses and outcomes on the other hand demand more focused policies, such as those related to health indicators and the need for information to enhance health outcomes. This study reveals that the greater an individual’s existing advantages, the more they benefit from the internet at a time of crisis; the converse is true as well. Marginalized people are likely to have fewer types of access available to take actions, behave as requested, or be comforted by help, creating a vicious cycle where already marginalized groups are further marginalized in a time of crisis.

To end on a positive note, the situation may become slightly less complex when we address the second goal of this paper. When people engage in information and communication internet uses in a crisis situation, their personal characteristics become less important to achieving the corresponding outcomes. This suggests that to achieve information and communication outcomes, policy or research should especially focus on encouraging people to engage in the corresponding internet uses, as we can assume to some extent that engagement with information and communication-related COVID-19 uses is the best way to achieve beneficial outcomes at a time when they are most needed.

### Limitations

The current study was conducted in the Netherlands, a country whose citizens have very high household internet penetration and high levels of educational attainment. Although differences in educational background and income are present and were taken into consideration, the observed inequalities may be even stronger in countries with a less homogeneous population. Given that the greatest burden of deaths has been in countries with very diverse populations, race and associated factors are likely to play a major role.

The aim of this study was to provide a broader picture of inequality in relation to how the internet is used in the case of a major global health crisis. A broad range of determinants was considered, and the relative importance of these indicators was revealed. However, a deeper understanding and further investigation to reveal the exact underlying mechanisms that cause these indicators to play a role would provide additional explanations. This suggests that further qualitative research is needed not only to obtain in-depth understanding of the mechanisms but also to understand the consequences of the observed inequalities to complement the findings of the current quantitative approach.

### Conclusions

Digital inequality is a major concern among national and international scholars and policy makers. In this paper, we aimed to provide a broader understanding in the case of a major health pandemic by using the ongoing COVID-19 crisis as a context for empirical work. Several groups of people were identified as vulnerable, such as older people and people with lower levels of education, physical health problems, higher levels of neuroticism, low literacy levels, and low levels of trust. The general conclusion is that people who are already relatively advantaged are more likely to use the information and communication opportunities provided by the internet to their benefit in a health pandemic, while more disadvantaged individuals are less likely to benefit. Therefore, the COVID-19 crisis is also an enforcer of existing inequalities.

## Abbreviations

COVID-19: coronavirus disease
MOS: Medical Outcomes Study
